# Complicated Case of Labor

**Published:** 1864-11

**Authors:** 


### SELECTED.

### COMPLICATED CASE OF LABOR.

#### PROLAPSUS OF THE FUNIS, ETC.

Dr. Alex. R. Simpson communicated to the Edinburgh Obstetrical Society, 9th of December, 1863, the following case, which presents some features of interest in respect, 1st, of the diagnosis of pregnancy; 2d, of the duration of pregnancy; 3d, of the painlessness of parturition; 4th, of prolapse of the umbilical cord, and its remedy by the postural treatment; 5th, of the employment of the forceps; and, 6th, of the management of the third stage of labor.

"1st, *Diagnosis of Pregnancy.*—Mrs. A. B. was in full process of parturition when she arrived at the Infirmary, on the 29th of November; but she had left home by the advice of her medical attendant, who saw her the night before, and told her she was laboring under a dropsical ovarian tumor. And yet nothing in obstetrics is more easy, when on our guard, than to distinguish between a gravid uterus at the full term, and a dropsical tumor of the ovary. There may be a common sensation of fluctuation, a common smoothness of surface, even a common situation in the abdominal cavity, but it needs only the application of the stethoscope to detect the distinct placental and cardiac sounds, or the touch of the finger, per vaginam, to recognize the hypertrophied uterus, with the moving body in the interior. In the present instance the mistake was made by a very able and accomplished practitioner, simply from his omitting duly to examine the case. He found the woman seated on a chair when he was summoned to see her, and, having already made up his mind that she could not be pregnant, he contented himself with putting his hand over the abdomen, and feeling the tumor through her dress. He then told her that she should go off to Edinburgh and have something done for it. And when she asked him for a line to secure her admission into the Infirmary, he added, that they were all interested in this kind of tumor at present, and that no recommendation was needed, for her case would commend itself. Now, the prejudice in his mind may have arisen from the circumstance, that when the patient, who is forty-four years of age, and the mother of seven children, had miscarried at the fifth month, two years ago, he had been led to form the opinion, which he then expressed to her, that she could never again become pregnant. But he was further led astray by the circumstance that more than ten months had elapsed since the date of her last menstruation; and it seems not to have occurred to him that the case might be, as it was, a case of

"2*d*, *Protracted Gestation.*—The patient menstruated in the beginning of January; but the period lasted only two days, instead of four as usual, having ceased on the 3d or 4th of the month. It was not till she felt "stirrage" in May, however, that she imagined herself to be pregnant, and began to think of making preparations for her confinement. She expected herself to have been delivered about the close of September or beginning of October, but it was not, as I have stated, till the end of November that the event took place. Supposing her not to have become impregnated till the period succeeding that at which the catamenia so abruptly ceased, she could not have carried the child less than 300 days; and if, as in the highest degree probable, both from the shortened menstruation and the date of quickening, she became impregnated at the period in January, we have a term of utero-gestation extending in this case to 329 days. The condition of of the child at birth, and especially of its head, indicated a corresponding degree of development. It measured 21 inches in length, and weighed over 10½ lbs. The head measured 14¾ inches in circumference; the anterior fontanelle was small, and the membrane firm; and the posterior fontanelle was so far obliterated, that the corner of the occipital could not be depressed below the parietal bones.

"3*d*, *Painless Parturition.*—Our patient having had all idea of pregnancy banished from her mind, set out from home, and reached the Infirmary without experiencing any uneasiness; and it was only while she was sitting in the nurse's room explaining the cause of her arrival, that a sudden gush of water from the vagina restored her to the conviction that she was, after all, to give birth to a child. The uterine contractions were going on, although she was unconscious of any pain; and they must have been going on for some time, for when the clinical clerk, Dr. Watson, saw her, almost immediately after the rupture of the membranes, he found the os uteri already fully dilated, and the head exposed. The woman had given birth to all her former children easily enough; but with all of them, she had, like other women, been conscious of the pain usually attendant on the uterine contractions. On this occasion, however, the first stage of labor was completed not only without any suffering having been experienced on her part, but also without her even being conscious that labor was in any degree in progress. And throughout the whole progress of the labor, the uterine contr'ctions seemed not to be accompanied with the slightest pain; for before the head entered into the pelvic brim, the patient was unconscious of any suffering, when the hand over the abdomen could feel the uterus distinctly contracting; and afterwards, when the head was passing through the pelvic canal, there was no kind of pain, only a feeling of fullness resulting from the pressure of the head on the soft parts of the pelvis. I had never before met with a case of absolutely painless parturition; but Dr. Von Ritgen, the venerable professor of midwifery, at Giessen, told me some years ago, that in the course of his practice he had seen seventeen women, who passed through the parturient process without any pain; and from his observation of these cases he had been led to form the conclusion that the act of parturition is normally and physiologically a painless one, which only becomes painful and pathological in consequence of the abnormal and artificial mode of life led by the great mass of womankind.

"*4th, Prolapsus Funis, and its Replacement by the Postural Treatment.*—I first saw the woman on going to visit, for my uncle, the patients in his ward in the Infirmary, at one o'clock, P. M., about a quarter of an hour after she had been seen by Dr. Watson. On making an examination, I found that a complication had occurred, from the falling down of a loop of the umbilical cord, of four or five inches in length, opposite the left sacro iliac synchondrosis. The umbilical vessels were pulsating vigorously, and the prolapsus must have taken place very shortly before, as there was none to be felt when Dr. Watson made the examination.

"We can easily understand the occurrence of prolapsus of the funis in cases of preternatural presentations and mal presentations of the head, or where, from contraction of the pelvic brim, the presenting part of the child is prevented from adapting itself closely to the lower segment of the uterus; but the conditions of its descent in cases of normal head-presentation have not yet been accurately ascertained.

"In the present instance, we have a concurrence of three of the conditions that have been more especially insisted on as favoring the occurrence of this accident. *First*, the patient was a multipara, with a very relaxed and dilatable cervix uteri and, perhaps, there was a want of tenacity in this part of the organ, associated in her with the absence of sensation during uterine action. *Secondly*, the placenta was placed very low down on the uterine wall, for the opening in the membranes was bounded in part by the placental margin; while, *thirdly*, the umbilical cord was inserted into the placenta within an inch of that part of its border;—two conditions, the importance of which have come to be abundantly acknowledged since the younger Naegele specially called attention to them, in an essay on the subject.* I might add, that the cord was of more than average length, and measured 21 inches, for this too has been noted in connection with prolapsus of the cord; but what exact share is to be attributed to each of these elements in the production of the complication, it would be difficult to decide, for it is a kind of case in which the mind of the accoucheur is for the time less taken up with the cause than with the cure; he is more anxious to avert the consequences of the accident, than to determine how it was produced.

"And here, let me observe, that the great variety of expedients that have been adopted for the remedy of this complication, and the vast variety of instruments that have been contrived for the reposition of the descended cord, are a sufficient indication of the imperfection and unsatifactoriness of each and all of them. And when we look to the recorded results of these various forms of treatment, and find that, even in the best hands, little more than two thirds of the children are saved, while the average mortality in the general mass involves more than half, we are prepared to welcome a suggestion so simple and safe, and to adopt a measure so satisfactory as that described in 1858, by Dr. T. G. Thomas, of New York, under the designation of 'The Postural Treatment.' * * Although six years have already elapsed since Dr. Thomas read his essay before the Academy of Medicine, the proposal is not widely enough known, or, at least, the results of the practice have not yet been recorded in sufficient abundance to allow us to make a statistical comparison of it with the multiform methods of treatment which it promises to replace. But I am well assured that when it shall come to be adopted as

* H. Fr. Naegele, Commentatio de causâ quâ tam prolap us funiculi umbilicalis in partu, non rarâ illâ quidem, sed minus notâ, Heidelb. 1839.

the common method of treating cases of prolapse of the cord, when interference is required, the high mortality of this complication will be found to be very materially diminished.

"In the special case before us the result was in every respect most gratifying. Having placed the patient on her elbows and knees, I passed the fingers of the right hand into the vagina, and carrying down the displaced loop of cord into the os uteri, I could feel it slip away past the presenting head into the dependent uterine cavity. Friction was then applied to the uterus to increase the contractions; and these having been still further stimulated by the administration of a full dose of ergot, and the head having fairly entered into the pelvic brim, the patient was made to resume the ordinary obstetric position in about a quarter of an hour from the commencement of the operation.

"5*th*, *Application of the Forceps.*—The uterus continued to contract regularly and steadily, though painlessly, but the advance of the head was so very slow, in consequence of its large size and extreme ossification, that after the lapse of an hour and a half, or two hours, I deemed it right to act on the principle, which has ever received the hearty sanction and support of this society, that we ought to interfere to avert the evils of delay, rather than to wait till nature has done her utmost, and left the patient prostrate, and, perhaps, after all, undelivered. The woman was accordingly brought under the influence of chloroform, when Dr. Watson applied the forceps and speedily effected her delivery. With the birth of the child in this manner, all peculiarity in the history o the case terminates. But I may still be permitted to add a few sentences as to the way in which we conducted,

"6*th*, *The Management of the Third Stage of Labor.*—With the left hand over the abdomen, I followed down the contracting uterus, as the body of the child was being expelled from its cavity; and then grasping the uterus, at first gently, then with more force, I compressed it until, within five or six minutes, the placenta, with the membranes, was driven into the vaginal orifice, and removed. At first sight it will be averred that there is nothing new in this kind of procedure; and it is not as a novelty in practice that I mention it now. Yet I apprehend that though a few practitioners in the midst of us are in the habit of following out this plan in most of their cases, the ordinary practice of the profession in Britain materially differs from it, and consists, as we find laid down in all our text-books, of waiting ten or fifteen minutes till the return of uterine contractions may have detached and expelled the placenta, then examining to discover the position of the afterbirth, and removing it; and when it does not come away at once, making gentle traction on the cord, while the uterus is stimulated to more energetic action by occasional friction, but not with such a forcible degree of compression as would suffice to separate and squeeze out the contents. Or if, in the practice of some obstetricians, the external manipulation of the uterus is insisted on as *the chief* element for successful completion of the third stage, yet even with them the internal interference with the cord and placenta is not entirely laid aside."—*Edinburgh Med. Journ.*, April, 1864.

